# Navigating the Complexity of Resistance in Lung Cancer Therapy: Mechanisms, Organoid Models, and Strategies for Overcoming Treatment Failure

**DOI:** 10.3390/cancers16233996

**Published:** 2024-11-28

**Authors:** Da Hyun Kang, Jisoo Lee, Subin Im, Chaeuk Chung

**Affiliations:** 1 Division of Pulmonology and Critical Care Medicine, Department of Internal Medicine, College of Medicine, Chungnam National University, Daejeon 34134, Republic of Korea; ibelieveu113@cnuh.co.kr; 2 College of Medicine, Chungnam National University, Daejeon 35015, Republic of Korea; jisoo98lee@gmail.com (J.L.);

**Keywords:** lung cancer, chemotherapy resistance, targeted therapy, immunotherapy, antibody–drug conjugate, targeted protein degradation, lung cancer organoid

## Abstract

**Background**: The persistence of chemotherapy-resistant and dormant cancer cells remains a critical challenge in the treatment of lung cancer. Objectives: This review focuses on non-small cell lung cancer and small cell lung cancer, examining the complex mechanisms that drive treatment resistance. **Methods**: This review analyzed current studies on chemotherapy resistance in NSCLC and SCLC, focusing on tumor microenvironment, genetic mutations, cancer cell heterogeneity, and emerging therapies. **Results**: Conventional chemotherapy and targeted therapies, such as tyrosine kinase inhibitors, often fail due to factors including the tumor microenvironment, genetic mutations, and cancer cell heterogeneity. Dormant cancer cells, which can remain undetected in a quiescent state for extended periods, pose a significant risk of recurrence upon reactivation. These cells, along with intrinsic resistance mechanisms, greatly complicate treatment efforts. Understanding these pathways is crucial for the development of more effective therapies. Emerging strategies, including combination therapies that target multiple pathways, are under investigation to improve treatment outcomes. Innovative approaches, such as antibody–drug conjugates and targeted protein degradation, offer promising solutions by directly delivering cytotoxic agents to cancer cells or degrading proteins that are essential for cancer survival. The lung cancer organoid model shows substantial promise to advance both research and clinical applications in this field, enhancing the ability to study resistance mechanisms and develop personalized treatments. The integration of current research underscores the need for continuous innovation in treatment modalities. **Conclusions**: Personalized strategies that combine novel therapies with an in-depth understanding of tumor biology are essential to overcome the challenges posed by treatment-resistant and dormant cancer cells in lung cancer. A multifaceted approach has the potential to significantly improve patient outcomes.

## Simple Summary

Chemotherapy-resistant and dormant cancer cells present significant hurdles in lung cancer treatment. Resistance to therapies such as chemotherapy and tyrosine kinase inhibitors is driven by factors including the tumor microenvironment and genetic mutations. Dormant cancer cells within minimal residual disease can evade detection, which contributes to cancer recurrence. Emerging strategies such as combination therapies, antibody–drug conjugates, and targeted protein degradation offer promising avenues to address these challenges. A deeper understanding of resistance mechanisms is crucial for the development of more effective, personalized treatment approaches. The lung cancer organoid model also holds immense potential to advance research and clinical applications in this field.

## 1. Introduction

Non-small cell lung cancer (NSCLC) and small cell lung cancer (SCLC) are the two primary types of lung cancer, each requiring distinct biological and therapeutic approaches. The initial treatment of metastatic NSCLC typically involves conventional chemotherapy combined with immune checkpoint inhibitors (ICIs) and targeted therapies such as tyrosine kinase inhibitors (TKIs) [1,2]. However, treatment failure and drug resistance remain significant challenges, and are driven by factors including tumor heterogeneity, physical barriers, dynamic genetic mutations, and immune system dysfunction [3]. Tumor heterogeneity results in varied responses to chemotherapy across different lesions, leading to poor long-term outcomes. Adaptive resistant subclones can survive chemotherapy, allowing their expansion [4,5,6]. Repeated biopsies of progressive or residual lesions and enhancing the precision of liquid biopsies are crucial strategies to address these challenges [7].

Dormant cancer cells, often undetectable on conventional computed tomography and positron emission tomography scans, play a key role in lung cancer progression and recurrence [8,9,10,11]. These dormant cells, referred to as minimal residual disease (MRD), represent a critical mechanism underlying lung cancer relapse. Cellular dormancy involves a quiescent state influenced by factors such as autophagy, stress-response pathways, microenvironmental signals, and epigenetic changes [12]. In clinical practice, salvage surgery or radiation therapy is actively pursued for lesions that either fail to respond to initial lung cancer treatment or represent MRD lesions that have remained stable but show signs of renewed growth. These interventions have demonstrated effectiveness in prolonging patient survival [13].

Current research aims to develop methods that effectively detect and eliminate dormant cells to improve treatment outcomes [14]. Ongoing studies also focus on the identification of triggers that reactivate dormant cells and the development of therapies to induce and maintain cancer cell dormancy [15].

Recent studies report that tumor heterogeneity leads to heterogeneous responses to treatment and therapy-persistent residual disease. The emergence and evolution of these cells are influenced by various factors, including the therapy-stimulated release of tumor secretome proteins, ATP release from dying cells, and the increased expression of APOBEC3A. To effectively target these persister cells, a clinical research framework should be developed that utilizes biomarkers, such as circulating tumor DNA (ctDNA) and cell-free DNA, to predict responses to standard care and incorporate persister-targeting agents into appropriate clinical trials [16].

SCLC is characterized by neuroendocrine features, rapid growth, and early metastasis [2,17]. Although immunotherapy has improved progression-free survival (PFS) and overall survival (OS) rates compared with conventional treatments, the prognosis remains poor. Resistance mechanisms in SCLC are associated with tumor heterogeneity, a low expression of major histocompatibility complex, and epigenetic changes, underscoring the need for novel therapeutic approaches [2,17].

Recent research has increasingly focused on lung cancer organoids (LCOs) derived from patient tissues for drug screening and use in personalized medicine. Although the creation of LCOs from standard biopsy samples initially posed challenges, advancements in techniques such as endobronchial ultrasound and cryobiopsy have significantly improved the LCO culture success rates from biopsy specimens [18,19,20,21]. This progress is expected to greatly enhance lung cancer research and patient care.

LCOs are actively used in studies of drug resistance, which provides valuable insights into chemotherapy resistance mechanisms and serves as a platform for the development of new therapies [22,23]. The use of LCOs offers great promise for understanding resistance pathways and advancing targeted treatment discovery [18,24,25].

This review explores the causes and mechanisms of treatment failure in both NSCLC and SCLC, discussing various strategies to address these challenges. It highlights the combination of existing treatments and the potential of emerging therapies, such as antibody–drug conjugates (ADCs) and targeted protein degradation, in combating drug resistance [26]. Additionally, this review showcases cutting-edge advancements in chemotherapy resistance research using LCOs.

## 2. Resistance Mechanism of Dormant Cancer Cells

**Figure 1:**
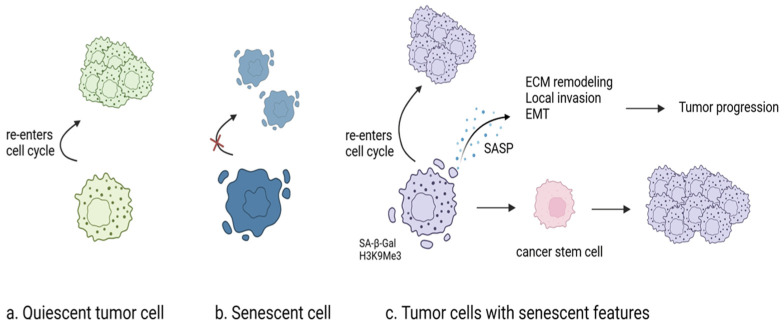
(**a**) Quiescent tumor cells can re-enter the cell cycle under favorable conditions. Reactivation can occur due to the activation of signaling pathways such as cGAS-STING, FAK, ERK, MLCK, YAP, and TGF-β, as well as neutrophil-mediated actions. (**b**) Senescent cells are generally unable to re-enter the cell cycle, as their arrest is irreversible. (**c**) Tumor cells with senescent features—either senescent-like tumor cells or senescent tumor cells—can facilitate tumor progression through various mechanisms. Senescent-like tumor cells may re-enter the cell cycle under appropriate conditions. In contrast, senescent tumor cells can promote local invasion, epithelial–mesenchymal transition (EMT), or extracellular matrix (ECM) remodeling by secreting SASP. Additionally, senescent tumor cells can acquire stemness and, in some cases, exhibit self-renewal activity. Created in BioRender. Im, S. (2024) https://BioRender.com/p43d923 (accessed on 28 November 2024).

Even after complete resection, the 5-year cumulative incidence of recurrence in patients with NSCLC was 20.1% [27]. Dormant cancer cells are one of the primary factors that account for the recurrence of lung cancer. The term dormancy encompasses a variety of concepts, from population-level dormancy to cellular dormancy. Population-level dormancy refers to a state in which a balance between cell division and cell death is obtained, keeping the cell population in an equilibrium [28]. Insufficient angiogenesis to support cancer cell proliferation or the elimination of malignant cells, such as NK cells and T cells, by the immune system accounts for the maintenance of this state [15,29]. In contrast, cellular dormancy refers to individual cells entering a reversible, quiescent state of growth arrest (G0-G1 arrest), allowing them to potentially resume division under suitable conditions [12,30,31]. However, classification of their dormancy level can often be vague, and each group is not mutually exclusive [8,11] (Figure 1).

Several recent studies have focused on the mechanisms in which cancer cell dormancy can remain unperturbed, offering new insights into the prevention of cancer recurrence by targeting specific signaling pathways. One of the pathways currently garnering attention is the cyclic GMP-AMP synthase-stimulator of interferon genes (cGAS-STING) pathway. When a ligand binds to and activates the STING pathway, various pro-inflammatory cytokines such as type I interferon are secreted, thereby recruiting immune cells that would suppress the metastasis [28]. In dormant and disseminated lung adenocarcinoma cells, activating the STING pathway innate to the cancer cell can prevent dormant cells from aggressively metastasizing [32]. Furthermore, STING agonists are known to increase the efficacy of conventional treatments, enabling them to overcome challenges related to treatment resistances [6]. For example, an orally available STING agonist was reported to increase the responsiveness of PD-1 blockade [32].

The FAK, ERK, MLCK, and YAP signaling pathways also play a role in the reactivation process of dormant cancer cells [33]. These pathways can be activated when sustained cellular stress such as lipopolysaccharide (LPS) or cigarette smoke induces the formation of neutrophil extracellular traps (NETs) [8,33]. However, another study documented that it is S100A8/A9 production by polymorphonuclear myeloid-derived suppressor cells (PMN-MDSCs), rather than NETs, that awakens dormant tumor cells through cellular stress [5]. On the other hand, the TGF-β pathway, along with the KLF10 positive feedback loop, activates cancer-associated fibroblasts (CAFs), thereby remodeling ECM and affecting cancer recurrence in lung adenocarcinoma [34]. Therapeutics targeting the above-mentioned signaling pathways can shed light on ways to prevent awakening the dormant lung cancer cells.

Senescent cells are distinct from quiescent cells in that their cellular arrest is irreversible, while quiescent cells can re-enter the cycle [35]. However, some dormant cancer cells render such distinctions meaningless as they can re-enter the cell cycle while sharing some phenotypes of senescent cells such as SA-β-gal and H3K9me3 [5,11]. For instance, surviving NSCLC cells treated with Osimertinib and Trametinib for combined EGFR/MEK inhibition primarily demonstrated SA-β-gal expression, indicating a senescent-like state. These senescent-like dormant cells were further characterized by YAP/TEAD signaling, suggesting this pathway as a critical pharmacological target for complete cancer eradication [11].

Notably, not all tumor cells with senescent markers can re-enter the cell cycle. Despite this, senescent tumor cells can still have pro-tumorigenic activities. For example, some senescent tumor cells can facilitate local invasion and remodel ECM by producing senescence-associated secretory phenotype (SASP) [12]. Others may promote EMT, increasing the metastatic potential [35].

Interestingly, these senescent tumor cells may also be related to cancer stem cells. When chemotherapy-induced senescent cells are reactivated, they tend to gain stem cell functions via activated Wnt and Notch signaling pathways [9,10,35]. As the cells gain stemness, their tumorigenicity increases dramatically, requiring initial detection and pharmacological therapies [10]. Whether the stemness of the cancer stem cells (CSCs) is therapy-induced or pre-existing, they pose significant barriers to cancer treatment. When treated with gefitinib, EGFR mutant lung cancer cells gained stemness attributes such as a self-renewal capability [36]. Similarly, gefitinib-resistant EGFR mutant NSCLC cells were shown to be high in stem cell markers such as CD133 and SOX2. In another case, the silencing of NOTCH signaling, which is one of the main three pathways (Wnt, Notch, and Hedgehog) in the development of lung stem cells, resulted in the regaining of gefitinib sensitivity and the reversal of the EMT phenotype. Therefore, efficient methods for monitoring CSC markers throughout the treatment process are in demand.

## 3. Mechanisms of Chemoresistance in NSCLC and Approaches to Overcome Conventional Treatment Failure

Treatment failure in NSCLC involves factors such as tumor heterogeneity, the undruggable genome, physical barriers, immune system dysfunction, tumor sanctuaries such as the brain, and the tumor microenvironment. Studies have shown that patients with significant tumor heterogeneity exhibit different responses to chemotherapy across lesions, leading to poor long-term outcomes [37,38].

Resistance mechanisms include modifications of cellular targets for chemotherapy, reduced intracellular drug levels, the inhibition of chemotherapy-induced cell cycle arrest and apoptosis, the acquisition of epithelial–mesenchymal transition and cancer stem cell-like traits, the dysregulation of microRNA expression, epigenetic alterations, and interactions with the tumor microenvironment [39]. The enhancement of liquid biopsy precision is essential for the early diagnosis of chemotherapy failure. Approaches to overcoming drug resistance include drug rechallenge, radiation therapy for oligometastasis, and anti-angiogenesis treatments.

After first-line treatment failure, approximately 25% of patients do not receive further chemotherapy. Common second-line monotherapies include docetaxel, pemetrexed, gemcitabine, and vinorelbine. Studies indicate that the continuation of chemotherapy after treatment failure improves patients’ quality of life compared with the best supportive care alone [40,41]. However, high doses of chemotherapy can cause severe side effects such as neutropenia, necessitating weekly regimens and careful dose adjustments. Cytotoxic chemotherapy is typically used after immunotherapy failure, although immunotherapy rechallenge or anti-angiogenesis agents such as bevacizumab and ramucirumab may also be considered [42,43]. Meta-analyses have shown that the reuse of previously administered immunotherapy is more effective and associated with fewer adverse events than a switch to a different immunotherapy type [44,45]. Rechallenge with immunotherapy has proven effective in patients who received immunotherapy for more than 2 years or discontinued it because of side effects. Even in those who previously experienced grade ≥2 adverse events, the occurrence of side effects tends to be delayed during rechallenge; nevertheless, significant adverse events such as arthritis, skin toxicity, colitis, and pneumonitis can still occur [45].

## 4. Resistance Mechanisms to TKIs in NSCLC: Challenges and Therapeutic Advances

TKI failures in patients with NSCLC can be classified as either on-target failures, which are due to resistance mutations in the target itself, or off-target failures, which result from alternative pathways or different genetic mutations. The main on-target resistance mechanisms for epidermal growth factor receptor (EGFR) TKIs include T790M and C797S mutations; the treatment strategies for EGFR-TKI failure vary based on whether the mutations are in cis or trans configurations [46,47]. Bypass mutations involving MET, ALK, RET, HER2, and HER3 require re-biopsy and testing with immunohistochemistry, fluorescence in situ hybridization, and next-generation sequencing [48,49]. Resistance to EGFR-TKIs can also lead to changes in the EGFR genetic profile, histological transformation into SCLC or squamous cell carcinoma, and alterations in the tumor immune microenvironment and programmed death-ligand 1 (PD-L1) expression [50,51]. ALK-related resistance mutations include L1196M, G1269A, and G1202R for crizotinib, with G1202R posing significant challenges for second-generation ALK inhibitors and, in combination with other mutations, for lorlatinib [52,53,54]. When considering re-biopsy for cancer resistance, factors such as potential subsequent treatments, the reuse of previous TKIs, and combination with local therapies must be carefully evaluated [55,56].

To prevent and overcome EGFR-TKI resistance, combinations of EGFR-TKIs with existing treatments such as platinum-based chemotherapy (PBC), mesenchymal–epithelial transition (MET) TKIs, and vascular endothelial growth factor receptor inhibitors can be effective. The FLAURA2 study demonstrated that patients with advanced and metastatic lung cancer harboring EGFR Ex19del/L858R mutations had significantly higher PFS and objective response rates when treated with osimertinib plus PBC than when treated with osimertinib alone, particularly in subgroups with L858R mutations and brain metastases [47,55,57]. However, no significant difference in patients’ OS was observed, and adverse events were substantially more common in the osimertinib plus PBC group. Thus, careful clinical consideration is required in these cases. The AGAIN study, which included three cycles of PBC during EGFR-TKI treatment to target resistant cells, did not show significant improvements in PFS or OS [55,58].

Amivantamab, a bispecific antibody that targets EGFR and MET and exhibits immune cell-directing activity, showed promise in the MARIPOSA study, where the combination of amivantamab with lazertinib resulted in increased PFS compared with osimertinib alone; the OS results are still pending [59]. However, adverse events in this study included a considerably higher incidence of venous thromboembolism in patients who received combination therapy than in those who received osimertinib alone (37% vs. 9%). Research is ongoing to compare savolitinib with osimertinib for patients with EGFR-TKI resistance and MET amplification or overexpression [60,61]. Comparisons of osimertinib plus bevacizumab with osimertinib alone have shown no significant differences in the PFS or OS, although there were higher incidences of hypertension and epistaxis in the combination group [62,63]. The RAMOSE study revealed a significantly longer PFS with ramucirumab plus osimertinib than with osimertinib alone (24.8 vs. 15.6 months, respectively) [64].

## 5. ICI Resistance in Lung Cancer: Mechanisms and Strategic Interventions

The use of ICIs as part of the first-line therapy for metastatic NSCLC and extensive-stage SCLC has become increasingly common in recent years. Although ICIs can result in durable responses in some patients, others may experience hyperprogression or severe, potentially fatal adverse events [65,66]. Therefore, careful consideration of the patient’s overall condition, underlying lung diseases—including interstitial lung disease—and tumor characteristics is essential when deciding whether to initiate immunotherapy [1].

Resistance to ICIs involves multiple immunological mechanisms, including T-cell exhaustion, impaired antigen recognition, defective T-cell migration and infiltration, and reduced T-cell cytotoxicity [67]. The tumor microenvironment significantly influences the efficacies and adverse effects of ICIs. Additional factors that contribute to ICI resistance include reduced antigen presentation, the production of neutralizing antibodies, the secretion of PD-L1 splicing variants, and influences from the gut and lung microbiota [68,69]. These factors must be addressed to optimize treatment outcomes and effectively manage risks [7,70]. The strategies to overcome resistance to ICIs include several promising approaches [71]. To address deficiencies in T-cell priming, combination therapies with radiotherapy, chemotherapy, or small-molecule inhibitors can be employed to enhance T-cell activation and priming [72]. To increase the number of tumor-reactive T cells, various strategies have been explored, such as adoptive cell transfer (ACT), including the transfer of tumor-infiltrating lymphocytes (TILs) and chimeric antigen receptor T cells (CAR-T cells) [73,74]. Additionally, approaches such as cancer vaccines, IL-15 cytokine therapies, and emerging agents like NC410 (a LAIR-2 Fc fusion protein) show potential in boosting the availability and functionality of tumor-specific T cells [75,76,77]. To reprogram or repolarize the immunosuppressive tumor microenvironment, therapeutic strategies which target key immune-regulatory pathways have been investigated. The use of IDO inhibitors has shown promise in mitigating the suppressive effects of metabolic pathways within the tumor microenvironment [78].

Ongoing clinical studies are exploring combination strategies to overcome immune checkpoint inhibitor (ICI) resistance in lung cancer [79]. Dual ICI blockade, such as the use of nivolumab plus ipilimumab, has demonstrated better overall and progression-free survival in patients with low or high PD-L1 expression, though toxicity remains a concern [80]. Novel agents like tiragolumab (anti-TIGIT), eftilagimod alpha (LAG-3), and utomilumab (4-1BB) are under investigation, with some showing enhanced anti-tumor responses in early trials [81,82]. Additionally, combining ICIs with VEGF inhibitors or chemotherapy has yielded improved survival in clinical trials, highlighting the potential of multi-targeted immunotherapy approaches. Radiotherapy combined with pembrolizumab has demonstrated superior outcomes in metastatic NSCLC patients, including a higher abscopal response rate (41.7% vs. 19.7%) and improved median overall survival (19.2 vs. 8.7 months), compared to pembrolizumab alone, as shown in pooled analyses of the PEMBRO-RT and MDACC trials [83,84]. Various clinical trials are investigating the use of antibody–drug conjugates (ADCs) combined with immune checkpoint inhibitors (ICIs), including atezolizumab with sacituzumab govitecan (MORPHEUS Lung) and pembrolizumab with trastuzumab deruxtecan, in lung cancer [85]. Triplet combinations of ADCs, ICIs, and chemotherapy are also being explored in trials such as the ADVANZAR, TROPION-Lung02, and EVOKE-02 trials for metastatic NSCLC [86]. Clinical trials are investigating the combination of ICIs with tumor-infiltrating lymphocyte (TIL) therapy in NSCLC. A phase I study combining nivolumab with TIL showed promising results, with 6 of 13 evaluable patients responding, including two with ongoing complete responses [87]. Other trials, such as the STARLING trial and those combining TIL with pembrolizumab or tislelizumab, are also underway, though some have faced challenges, including increased toxicities in certain regimens [79]. Ongoing clinical trials are exploring neoantigen-directed vaccines combined with immune checkpoint inhibitors (ICIs), including EVAX-01-CAF09b, NEO-PV-01, PANDA-VAC, and PNeoVCA, in lung cancer [69,88]. These personalized vaccines target tumor-specific neoantigens and are being evaluated with pembrolizumab and chemotherapy in various settings, although results have not yet been reported.

Relative to conventional chemotherapy and targeted therapy, ICIs are associated with higher incidences of fatal adverse effects and hyperprogression. Thus, although overcoming resistance is essential, clinicians must carefully consider whether to initiate immunotherapy [7]. Recent studies have explored ways to better predict the efficacies and adverse effects of ICIs. These efforts include the analysis of patients’ immune profiles at the single-cell level and the utilization of artificial intelligence to assess biomarkers such as the patient’s tumor mutational burden, PD-L1 tumor proportion score, history of systemic therapy, blood albumin level, neutrophil-to-lymphocyte ratio, age, sex, and cancer type [89,90]. These advances aim to refine patient selection, ensuring improved outcomes and minimizing the risks of severe side effects.

## 6. Innovative Therapeutics: ADCs and Targeted Protein Degradation

Research is ongoing to overcome EGFR-TKI resistance using ADCs, which consist of antibodies, payloads, and linkers [91] (Table 1). Anti-human epidermal growth factor receptor 2 (anti-HER2) drugs, such as trastuzumab emtansine and trastuzumab deruxtecan, are under investigation for patients with HER2 mutations [26]. HER3 is expressed in approximately 80% of NSCLC and from 85% to 100% of cancers with EGFR mutations. Patritumab deruxtecan, which targets HER3, demonstrated an objective response rate of 39% and a median PFS of 8.2 months in EGFR-TKI–resistant patients [92]. MET-targeting agents include telisotuzumab vedotin, which is being studied in combination with osimertinib and docetaxel. Additionally, datopotamab deruxtecan and sacituzumab govitecan are under evaluation for targeting trophoblast cell-surface antigen 2, whereas tusamitamab ravtansine targets carcinoembryonic antigen-related cell adhesion molecule 5 [91].

Recent research has shifted toward innovative approaches to overcoming cancer drug resistance, particularly focusing on targeted protein degradation, a novel therapeutic strategy that selectively degrades specific proteins critical for cancer cell survival. This method aims to eliminate drug-resistant cancer cells more effectively, providing a promising alternative to conventional therapies that often lose efficacy due to resistance mechanisms. Targeted protein degradation involves the use of structures that link E3 ligase recognition motifs to target protein ligands [93]. Two main strategies involve the use of molecular glues, which directly bind target proteins to ubiquitin ligase, and proteolysis-targeting chimeras (PROTACs), which possess dual-functional domains that bind to both target proteins and ubiquitin ligase [94,95]. EGFR degraders that utilize PROTACs are currently being studied to overcome osimertinib resistance [95,96].

Several PROTAC molecules that target key oncogenic drivers in NSCLC have been developed, such as EGFR, KRAS, ALK, BRAF, and BCL-XL. These compounds have shown significant antitumor activity in both cellular and preclinical tumor models, suggesting their potential as a new class of therapeutics for NSCLC. Unlike small-molecule inhibitors, PROTACs can degrade oncogenic proteins, including those with resistant mutations, at minimal intracellular concentrations [96]. Each PROTAC molecule can catalyze multiple degradation cycles, allowing the efficient and sustained removal of target proteins (Table 2). This catalytic property offers an advantage over conventional inhibitors, providing a novel mechanism to overcome resistance associated with conventional therapies. The targeted degradation of validated proteins highlights the potential of PROTACs in future cancer treatment strategies [94]. 

**Table 1:** Examples of ADCs and TPDs with key features and FDA Approval Status for NSCLC.

Treatment Strategy	Target	Drug/Compound	Key Features	Fda Approval Status	Reference
ADC	HER2	Trastuzumab emtansine	Has shown efficacy in HER2-positive breast cancer	Not approved for NSCLC	[91]
ADC	HER2	Trastuzumab deruxtecan	ORR 55%, median PFS 8.2 months in HER2-mutant NSCLC	Approved for HER2- mutant metastatic NSCLC (2022)	[97]
ADC	HER3	Patritumab deruxtecan	ORR 39%, median PFS 8.2 months in EGFR-TKI-resistant patients	Not approved	[92]
ADC	MET	Telisotuzumab vedotin	Under evaluation with osimertinib and docetaxel	Not approved	[98]
ADC	TROP2	Sacituzumab govitecan	Has shown good clinical activity in most solid tumor types	Not approved for NSCLC	[85]
TPD	EGFR*^Del19^*, EGFR^L858R/T790M^	PROTAC EGFR degraders	Cellular and preclinical antitumor activity	Not approved	[99]
	KRAS*^G12C^*	PROTAC KRAS degraders		Not approved	
	ALK	PROTAC ALK degraders		Not approved	
	BRAF*^V600E^*	PROTAC BRAF degraders		Not approved	
	BCL-XL	PROTAC BCL-XL degraders		Not approved	

**Table 2:** Comparison of Mechanisms and Examples of Targeted Protein Degraders.

Targeted Protein Degrader	Mechanism	Examples of Target Protein	Antitumor Activity	Reference
Molecular Glues	Directly bind target protein to E3 ligase	GSTP1, IKZF1/3	Effective in hematologic cancers, under evaluation in NSCLC.	[94,96,100]
PROTACs	Possess dual-functional domains that bind both target protein and E3 ligase	EGFR*^Del19^*, EGFR^L858R/T790M^, KRAS*^G12C^*, ALK, BRAF*^V600E^*, BCL-XL	Significant in both cellular and preclinical tumor models	[51,52,53,54,99]

## 7. Underlying Mechanisms of Therapeutic Resistance in SCLC

SCLC is a highly aggressive malignancy characterized by rapid progression, high relapse rates, and significant morbidity and mortality rates [84]. Although the immunotherapy for extensive-stage SCLC has shown improved outcomes, with a PFS of 5–6 months and OS of 12–15 months compared with conventional therapies, the overall prognosis remains poor [101,102,103]. SCLC initially responds well to cytotoxic chemotherapy, but resistance typically develops rapidly, leading to a poor prognosis. Understanding the molecular mechanisms underlying this resistance is critical for developing new therapeutic strategies.

Genetic mutations play a central role in the development of chemotherapy resistance in SCLC. Mutations in tumor suppressor genes such as TP53 and RB1 are nearly universal in SCLC and are associated with poor responses to treatment. These mutations disrupt the normal cell cycle control and DNA damage response, allowing cancer cells to survive despite chemotherapy-induced DNA damage [104]. With advancements in techniques for profiling cancer genomes and transcriptomes, recent studies have provided deeper insights into the heterogeneity of SCLC. Recent strategies to overcome drug resistance include the combination of thoracic radiotherapy, even in extensive-stage disease, with other therapies. SCLC can be classified into neuroendocrine subtypes (SCLC-A, SCLC-N) and non-neuroendocrine subtypes (SCLC-Y, SCLC-P) based on its expression of ASCL1, NEUROD1, POU2F3, and YAP1; SCLC-A represents 50–70% of cases [104]. Additionally, a newly identified immune-related, inflammatory subtype (SCLC-I), which lacks ASCL1, NEUROD1, and POU2F3 but has a high expression of immune-related proteins, has been introduced [104]. Ongoing research is targeting key pathways associated with SCLC, including the Notch, DLL3, MYC, mTOR, and BCL2 pathways [105,106]. Since the characteristics that contribute to treatment resistance differ across these SCLC subtypes, distinct therapeutic strategies can be considered for each subtype [106].

Proteomic analyses of SCLC cell lines have identified elevated levels of DNA repair proteins, such as PARP1, ATM, CHK1, and EZH2 [90]. DNA damage repair includes mechanisms such as DNA double-strand break repair, base excision repair, nucleotide excision repair, and mismatch repair, all of which are known to significantly influence patients’ drug response [107]. Comprehensive bioinformatics analyses have revealed that adherent or semi-adherent SCLC cells exhibit increased activity of the PI3K/Akt/mTOR pathway and show significant resistance to chemotherapy [108]. Activation of the Kras or Notch pathways triggers a transition from neuroendocrine to non-neuroendocrine cell fates, contributing to increased chemotherapy resistance in SCLC [106]. MYC facilitates the evolution of SCLC subtypes by reprogramming neuroendocrine cells via Notch pathway activation, further promoting chemoresistance [109]. Additionally, EZH2 is upregulated in chemo-resistant SCLC and enhances drug resistance by epigenetically silencing Schlafen family member 11 (SLFN11) [110]. Apoptosis, a form of programmed cell death, plays a crucial role in patients’ chemotherapy response, and, in SCLC, resistance can develop due to disruptions in the apoptotic machinery. BCL-2, a key regulator of apoptosis, is highly expressed in many SCLC tumors, and several studies suggest synergy between the inhibition of PI3K–mTOR and BCL-2 in SCLC [111]. Various clinical studies are underway that target pathways highly associated with SCLC, including the Notch, DLL3, MYC, mTOR, and BCL2 pathways [112].

The advent of immunotherapy, specifically PD-1/PD-L1 inhibitors, has provided new hope for SCLC patients. However, the efficacy of these therapies is often limited by the development of resistance. Understanding the mechanisms underlying resistance to PD-1/PD-L1 inhibitors is essential for improving patient outcomes.

The tumor microenvironment (TME) in SCLC is highly immunosuppressive, which facilitates immune evasion. SCLC tumors often upregulate immunosuppressive molecules such as TGF-β and IL-10, which inhibit the activity of cytotoxic T cells and promote the development of regulatory T cells (Tregs). These factors create a hostile environment for immune cells, reducing the efficacy of PD-L1 inhibitors [113]. An additional immune evasion mechanism involves the downregulation of MHC class I and II molecules, leading to impaired antigen presentation. Notably, SCLC exhibits low intrinsic levels of MHC class I molecules, including HLA-A, HLA-B, HLA-C, and β2-microglobulin, along with several genes associated with MHC-mediated antigen presentation. This results in diminished neoepitope presentation to CD8+ T cells and the reduced recognition of tumor neoantigens by cytotoxic T cells [114]. In addition, PD-L1 is not the only immune checkpoint that can be targeted by cancer cells to evade their destruction by the immune system. The high expression of B7-H3, a ligand from the B7 family known to facilitate various pro-tumorigenic and immunosuppressive functions, has been observed in SCLC specimens, correlating with a decreased infiltration of lymphocytes within the tumor microenvironment [115]. Based on these findings, several clinical trials are investigating novel potential targets to inflame the TME of ICI-resistant SCLC.

## 8. Strategies to Overcome Treatment Resistance in SCLC

Treatment resistance in SCLC remains a significant challenge, with many patients experiencing relapse despite their initial responsiveness to therapy. To address this, researchers have focused on developing strategies that target the underlying mechanisms of resistance, aiming to improve patient outcomes. Herein, we describe some of the most promising approaches currently under investigation, with a focus on recent studies.

Lurbinectedin, a new second-line drug for SCLC, is currently in use, and new agents such as bispecific T-cell engagers and PARP inhibitors are under investigation [116,117]. Lurbinectedin, an alkylating agent, induces DNA damage, inhibits RNA polymerase II, and modulates the tumor microenvironment by inhibiting tumor-associated macrophages [117,118]. In a phase II clinical trial, lurbinectedin showed significant activity in patients with relapsed SCLC, leading to its accelerated FDA approval [118]. Current research is focused on combining lurbinectedin with other agents, such as immunotherapies and additional chemotherapy drugs, to overcome resistance and improve outcomes in SCLC patients [119]. Delta-like ligand 3 (DLL3), which is regulated by ASCL1 and inhibits Notch signaling, is highly expressed in SCLC and is a target in ongoing research that aims to overcome resistance to existing chemotherapy [109,120,121] (Table 3).

T-cell engager molecules physically link immune cells and cancer cells by binding to T-cell receptors and target antigens on cancer cells. Among these, tarlatamab, a bispecific T-cell engager that targets DLL3 and CD3, is in development [122]. Preclinical studies have shown promising results when bispecific T-cell engagers are combined with immunotherapy. SCLC is characterized by high levels of genomic instability, making DNA damage repair pathways attractive targets for overcoming drug resistance in SCLC. PARP inhibitors, such as olaparib and talazoparib, which disrupt the DNA damage response, have demonstrated efficacy in SLFN1-positive SCLC; combination strategies that induce synthetic lethality are under active exploration [70,73]. SCLC proliferation is closely linked to microvessel formation, which leads to the overexpression of VEGF, which is associated with a poor prognosis [123]. Targeting angiogenesis may represent a promising therapeutic approach to SCLC, and the effectiveness of the anti-VEGF antibody bevacizumab, along with small-molecule tyrosine kinase inhibitors (TKIs) aimed at the VEGF receptor (VEGF-R), has been explored in various studies [124].

Combining immune checkpoint inhibitors with other therapeutic agents has been a critical strategy in overcoming resistance in SCLC. Recent studies have explored combining immune checkpoint inhibitors with other agents, such as anti-angiogenic drugs and PARP inhibitors, to enhance the anti-tumor response and delay resistance of patients [116,122]. Moreover, numerous studies have indicated that radiotherapy (RT) has an immunomodulatory effect that could potentially improve the efficacy of immune checkpoint inhibitors (ICIs) [125]. Historically, RT for extensive-stage small cell lung cancer (ES-SCLC) was primarily used for palliative care; however, in the context of chemo-immunotherapy, the role of consolidative thoracic RT for patients with ES-SCLC is currently being explored in several clinical trials [114].

These approaches are particularly relevant when adjunctive therapies are considered in combination with ICIs to enhance the likelihood, duration, and effectiveness of anti-tumor responses.

A recent study demonstrated the successful culture of SCLC organoids for drug screening [126,127]. These organoids offer promise for comprehensive subtype analyses and investigations into drug resistance, providing a foundation for more effective therapeutic strategies against SCLC.

**Table 3:** Strategies to overcome treatment resistance in SCLC.

Strategy	Treatment	Mechanism	Clinical Evidence	Reference
Alkylating agent	Lurbinectedin	Induces DNA damage, Inhibits RNA polymerase II, Modulates tumor microenvironment	Phase II trial: Significant activity in relapsed SCLC, FDA accelerated approval Ongoing research combining lurbinectedin with immunotherapies and chemotherapy	[118]
Bispecific T-cell Engagers	Tarlatamab (DLL3/CD3)	Binds T-cell receptors and cancer cell antigens, linking immune cells to tumors	*n* patients with heavily pretreated SCLC, tarlatamab demonstrated manageable safety with encouraging response durability	[128]
PARP inhibitors	Olaparib, Talazoparib	Disrupts DNA damage repair mechanisms	Efficacy in SLFN1-positive SCLC; combination strategies under investigation	[129]
Angiogenesis inhibition	Bevacizumab (anti-VEGF antibody), VEGF receptor inhibitors (TKIs)	Targets microvessel formation and VEGF overexpression	Clinical trials exploring combination with other agents for improved efficacy	[124]
Combination immunotherapy	Immune checkpoint inhibitors (ICIs) + PARP inhibitors, ICI + Anti-angiogenic agents	Enhances anti-tumor immune response and delays resistance	Ongoing studies: Investigating combinations with PARP inhibitors, anti-VEGF agents	[130]
Radiotherapy	Consolidative thoracic radiotherapy (RT) with chemo-immunotherapy	Immunomodulatory effects that may enhance ICI efficacy	Clinical trials: Exploring RT as part of chemo-immunotherapy in ES-SCLC	[131]

## 9. Harnessing LCOs to Address Drug Resistance and Optimize Treatment

LCOs are considerably more challenging to create than organoids from other cancers [23]. One of the primary challenges is the invasive nature of lung tissue sample acquisition through percutaneous needle biopsies, which carry significant risks such as pneumothorax, hemothorax, and potentially fatal complications [132,133,134]. This makes lung tissue biopsy considerably more dangerous than breast or thyroid cancer biopsies. Additionally, unlike gastrointestinal cancers such as stomach and colorectal cancers, where tumors are located in the hollow viscera, access to lung cancer tissue requires navigation through a branching, progressively narrowing bronchial system, making the procedure technically difficult [18,135] (Table 4).

As a result, the amount of tissue obtained from lung biopsies is often limited, and repeat biopsies are more challenging. However, recent advances, including endobronchial ultrasound and cryobiopsy techniques, have allowed for the safer and more substantial collection of lung cancer tissue [18,19,135]. These innovations have enhanced lung cancer diagnostics while facilitating significant breakthroughs in the development and culture of LCOs.

Studies have been conducted to establish an LCO (lung cancer organoid) bank to verify that LCOs accurately recapitulate the characteristics of patients’ primary tissues. Additionally, research has been aimed at optimizing the composition of organoid culture media to prevent the overgrowth of normal cells [19].

To prevent normal lung cells from growing alongside LCOs or overgrowing, some studies have created LCOs using samples obtained from malignant pleural effusions or metastatic lesions. However, a limitation of this approach is that not all patients have malignant pleural effusions or biopsy-accessible metastatic lesions [135].

Wang et al. presented findings on the clinical use of patient-derived lung cancer organoids (LCOs) to predict the tumor response in cases of locally advanced or metastatic lung cancer. Their findings indicate that lung cancer organoids (LCOs) derived from malignant effusions have a success rate exceeding 80%, with LCO-based drug sensitivity assays effectively predicting clinical responses to treatment [136].

Recently, studies utilizing 3D holotomography have enabled the detailed visualization of live organoids in 3D, offering promising advancements in the analysis of lung cancer organoids [137].

Research using LCOs for drug screening and to overcome resistance is rapidly advancing. Co-culture systems that include fibroblasts and immune cells are in development to better mimic the tumor microenvironment, enhancing the relevance of LCO models [138,139]. Additionally, recent studies have explored the use of 3D printing technologies to create “organ-on-a-chip” models that more accurately replicate in vivo conditions of lung cancer, thus providing new insights into disease mechanisms and treatment strategies [25,140] (Table 5).

**Table 4:** Studies on drug screening using lung cancer patient-derived organoids (PDOs).

Topic of Study	Drug/Targets	Outcome	Reference
Establishment of PDO biobank from 10 NSCLC patients for drug screening	Natural compounds (chelerythrine chloride, cantharidin, harmine, berberine, betaine)	Identified anticancer activity of natural compounds (chelerythine chloride, cantharidin, harmin) on both PDOs and cell lines.	[127]
Validation of NSCLC PDOs for predicting treatment response	26 anti-cancer drugs	PDOs retained the histological and genetic characteristics of the primary tumors. The in vitro response to drug screening with PDOs highly correlated with the mutation profiles in the primary tumor.	[141]
Evaluation of anticancer agents using PDOs with squamous and adenosquamous lung cancers	78 anticancer agents including molecular target drugs, immune-checkpoint inhibitors	In vitro assay systems using PDOs were suitable for evaluating molecular targeted drugs.	[142]
Establishment of biobank of 80 LCOs for drug screening	Targeted therapies including erlotinib, crizotinib, olaparib	Drug responses in LCOs correlated with genomic alterations	[19]
Establishment of 12 PDOs from lung adenocarcinoma	24 anti-cancer drugs	The drugs with the same targets displayed reproducible sensitivity patterns among organoid lines	[143]

**Table 5:** Registered clinical trials using patient-derived lung cancer organoids for drug response prediction.

Topic of Study	Study Type	Outcome Measures	Status/Location	Reference
Predict the therapeutic response using PDOs in patients with multi-line drug-resistant lung cancer	Interventional	Correlation of ex vivo drug sensitivity tests on PDO with clinical outcomes	Recruiting/Affiliated Hospital of Jiangnan University	NCT05669586
PDOs of lung cancer to test drug response	Observational	PDO establishment and validation Prediction of the response to treatment by the PDOs	Recruiting/Geneva University Hospitals	NCT03979170
PDO drug sensitivity guided treatment for recurrent SCLC	Interventional	Statistical analysis on the consistency between drug sensitivity test results and patient treatment response	Recruiting/Henan Cancer Hospital	NCT06406660
PDO drug sensitivity guided treatment for drug-resistant recurrent NSCLC	Interventional	Statistical analysis on the consistency between drug sensitivity test results and patient treatment response	Recruiting/Henan Cancer Hospital	NCT06406608
Evaluation of the response to tyrosine kinase inhibitors in NSCLC patients with EGFR mutation in a PDO model	Observational	Evaluation of the in vitro efficacy of osimertinib in a patient-derived organoid model alone or in combination	Unknown/Central Hospital, Nancy, France	NCT05136014

## 10. Conclusions

Drug resistance remains a significant challenge in lung cancer treatment, necessitating ongoing research and innovative approaches. A comprehensive analysis of the tumor heterogeneity and dormant cells within minimal residual disease is critical, highlighting the urgent need to develop therapies that effectively target these elements to improve treatment outcomes. This review has explored the causes and mechanisms of treatment failure in NSCLC and SCLC, proposing various strategies to address these challenges. It underscores the importance of understanding resistance mechanisms to chemotherapy and TKIs; it also highlights strategies such as the use of liquid biopsies, drug rechallenge, radiation therapy, and anti-angiogenesis treatments.

This review also emphasizes the potential of combination therapies and novel approaches, such as the use of ADCs and PROTACs, to overcome EGFR-TKI resistance. It presents innovative strategies for treating SCLC, including the use of bispecific T-cell engagers and PARP inhibitors, underscoring the importance of tailored therapeutic approaches. The LCO model has emerged as a valuable tool for studies of chemotherapy resistance, offering significant clinical benefits by supporting the development of personalized treatments and improving patient outcomes.

To effectively overcome drug resistance in lung cancer, personalized treatment strategies that consider the heterogeneity of tumors and the continued development and clinical application of new therapies are essential. This review aims to contribute to further research and clinical advancements in the field, ultimately enhancing patient care and outcomes.

## Abbreviations

NSCLC: Non-Small Cell Lung Cancer, SCLC: Small Cell Lung Cancer, ICIs: Immune Checkpoint Inhibitors, TKIs: Tyrosine Kinase Inhibitors, MRD: Minimal Residual Disease, PFS: Progression-Free Survival, OS: Overall Survival, LCOs: Lung Cancer Organoids, ADCs: Antibody–Drug Conjugates, EGFR: Epidermal Growth Factor Receptor, MET: Mesenchymal–Epithelial Transition, ALK: Anaplastic Lymphoma Kinase, RET: Rearranged During Transfection, HER2: Human Epidermal Growth Factor Receptor 2, HER3: Human Epidermal Growth Factor Receptor 3, PD-L1: Programmed Death-Ligand 1, PD-1: Programmed Death-1, PBC: Platinum-Based Chemotherapy, CT: Computed Tomography, PET: Positron Emission Tomography, MHC: Major Histocompatibility Complex, VEGF: Vascular Endothelial Growth Factor, VEGF-R: Vascular Endothelial Growth Factor Receptor, PROTACs: Proteolysis-Targeting Chimeras, SLFN11: Schlafen Family Member 11, DLL3: Delta-Like Ligand 3, TME: Tumor Microenvironment, Tregs: Regulatory T Cells, RT: Radiotherapy, DNA: Deoxyribonucleic Acid, PARP: Poly (ADP-Ribose) Polymerase, EZH2: Enhancer of Zeste Homolog 2, PI3K: Phosphoinositide 3-Kinase, Akt: Protein Kinase B, mTOR: Mammalian Target of Rapamycin, HLA: Human Leukocyte Antigen, KRAS: Kirsten Rat Sarcoma Viral Oncogene Homolog, BRAF: B-Raf Proto-Oncogene, BCL-XL: B-Cell Lymphoma Extra Large, ASCL1: Achaete-Scute Family BHLH Transcription Factor 1, NEUROD1: Neuronal Differentiation 1, POU2F3: POU Class 2 Homeobox 3, YAP1: Yes-Associated Protein 1, FDA: Food and Drug Administration, Ex19del/L858R: Specific Mutations in the EGFR Gene, FISH: Fluorescence In Situ Hybridization, NGS: Next-Generation Sequencing, BCL-2: B-Cell Lymphoma 2, SLFN1: Schlafen Family Member 1, IL-10: Interleukin-10, TGF-β: Transforming Growth Factor Beta, CD3: Cluster of Differentiation 3, CD8+: Cluster of Differentiation 8 Positive, MYC: Myelocytomatosis Oncogene, ATM: Ataxia Telangiectasia Mutated, CHK1: Checkpoint Kinase 1, E3: Ubiquitin-Protein Ligase E3, PET: Positron Emission Tomography, CTLA-4: Cytotoxic T-Lymphocyte–Associated Protein 4, ES-SCLC: Extensive-Stage Small Cell Lung Cancer, B7-H3: CD276 Molecule, CD8+ T Cells: Cytotoxic T Lymphocytes, MARIPOSA Study: A Clinical Trial Evaluating Amivantamab with Lazertinib, FLAURA2 Study: A Clinical Trial Evaluating Osimertinib Plus Platinum-Based Chemotherapy, AGAIN Study: A Clinical Trial Evaluating Platinum-Based Chemotherapy During EGFR-TKI Treatment, RAMOSE Study: A Clinical Trial Evaluating Ramucirumab Plus Osimertinib.
